# Phylogeographical Analysis of mtDNA Data Indicates Postglacial Expansion from Multiple Glacial Refugia in Woodland Caribou (*Rangifer tarandus caribou*)

**DOI:** 10.1371/journal.pone.0052661

**Published:** 2012-12-21

**Authors:** Cornelya F. C. Klütsch, Micheline Manseau, Paul J. Wilson

**Affiliations:** 1 Biology Department, Trent University, Peterborough, Ontario, Canada; 2 Northern and Western Service Centre, Parks Canada, Winnipeg, Manitoba, Canada; 3 Natural Resources Institute, University of Manitoba, Winnipeg, Manitoba, Canada; University of York, United Kingdom

## Abstract

Glacial refugia considerably shaped the phylogeographical structure of species and may influence intra-specific morphological, genetic, and adaptive differentiation. However, the impact of the Quaternary ice ages on the phylogeographical structure of North American temperate mammalian species is not well-studied. Here, we surveyed ∼1600 individuals of the widely distributed woodland caribou (*Rangifer tarandus caribou*) using mtDNA control region sequences to investigate if glacial refugia contributed to the phylogeographical structure in this subspecies. Phylogenetic tree reconstruction, a median-joining network, and mismatch distributions supported postglacial expansions of woodland caribou from three glacial refugia dating back to 13544–22005 years. These three lineages consisted almost exclusively of woodland caribou mtDNA haplotypes, indicating that phylogeographical structure was mainly shaped by postglacial expansions. The putative centres of these lineages are geographically separated; indicating disconnected glacial refugia in the Rocky Mountains, east of the Mississippi, and the Appalachian Mountains. This is in congruence with the fossil record that caribou were distributed in these areas during the Pleistocene. Our results suggest that the last glacial maximum substantially shaped the phylogeographical structure of this large mammalian North American species that will be affected by climatic change. Therefore, the presented results will be essential for future conservation planning in woodland caribou.

## Introduction

Geographical species ranges and intra-specific genetic diversity are frequently closely linked to the cyclic climatic changes of the Quaternary [1]–[5] including the persistence of species subgroups in refugia during glaciation from which they expanded after glacial periods upon retreat of ice sheets [1]–[7]. The identification and dating of such glacial refugia has been an active field of research [7], [8] providing opportunities to study evolutionary processes like genetic diversification, adaptation, speciation, and extinction events [3], [4]. Studying the impact of glacial refugia on a species’ genetic diversity may also offer information about the impact of forthcoming climatic changes on contemporary living species [2]–[5]. Hence, conservation efforts may greatly benefit from an increased understanding of past species responses to climatic changes and this in turn may explain intra-specific morphological, genetic, ecological differentiation, and local adaptation in contemporary species [3], [5], [9]. Importantly, species responded differently to climate change [3]–[5], [7], [10] and therefore, comprehensive case studies are required to determine species-specific phylogeographical structure due to glacial refugia and implement this knowledge into conservation efforts associated with adaptive potential.

The last continental glacial maximum (26500–19000 calendar years before present (YBP) [11] in North America led to the formation of a massive ice sheet (Laurentide ice sheet) that covered most of its northern land mass and extended south to about 39°N [10], [12] (Figure S1). As a result, numerous North American animal and plant taxa were likely distributed south of the ice sheet during the glacial maximum and consequently, a lot of today’s northern biodiversity is largely derived from geographically restricted ancestral populations in these southern glacial refugia [10]. However, the impact of glacial cycles on North American mammalian species has only been studied in a few cases so far [10] and more comprehensive studies are needed to complement our knowledge of how the last glacial maximum shaped phylogeographical structure in large temperate North American mammals. This is of special importance because most of these large mammal species will be affected by climate change. Several major glacial refugia have been identified for both animal and plant species in North America [10], [13] south of the Laurentide ice sheet. Based on two meta-analyses [10], [13] these refugia seem to be in the Gulf of Mexico coastal region separated by the Mississippi River and the Apalachicola River /Appalachian mountain system as well as south of the Appalachian Mountains in Eastern North America and the big mountain chains (e.g., Rocky Mountains) and adjacent areas along the western coast line of North and Central America.

The North American caribou (*Rangifer tarandus*) is widely distributed throughout Canada/Alaska and presents a model exemplifying different layers of morphological, genetic, and ecological differentiation. Within its distribution range (Figure S1), five native subspecies are currently recognized [14]–[18]: Dawson’s caribou (*R. t. dawsoni*, extinct), barren-ground caribou (*R. t. groenlandicus*), woodland caribou (*R. t. caribou*), Grant’s caribou (*R. t. granti*), and Peary caribou (*R. t. pearyi*). Of these, woodland caribou is of special significance for conservation biologists and policy makers, because about half of the ‘populations’ (i.e. designatable units (DUs), herds, and aggregations) are decreasing or are in one of the “at risk” categories (e.g. ‘threatened’, ‘endangered’, or ‘of special concern’ [17]–[19]. Increasing human-induced habitat disturbance and loss [20]–[25] and changing predator-prey dynamics [26], [27] have been identified to different degrees as current threats for woodland caribou. The potential effect of global warming on this wide-ranging species has also been discussed as an additional current and future hazard [25], [28], [29].

Woodland caribou present considerable morphological, genetic, and behavioural variability reflective of both plasticity and local adaptation which has recently resulted in the identification of 8 designatable units (DUs) in the subspecies’ range [18]. One potential source of this variability could be the evolution of diverse lineages that originated from multiple glacial refugia. The fossil record supports at least four northern glacial refugia in Beringia, northeastern Greenland, in areas south of the Cordilleran and Laurentide ice sheet, and there is evidence of a refugium on Banks Island [30], [31] for caribou [32]. In contrast, the temperate woodland caribou most likely originated from south of the Laurentide ice sheet [14], [32]–[34] whereas the other four (arctic/tundra) subspecies originated in northern refugia; possibly explaining part of the differentiation at the subspecies level. Previous genetic studies [34], [35] suggested that there was at least one major well-defined refugial area in North America for the woodland caribou south of the Laurentide ice sheet. Also, it has been indicated by a multimodal mismatch distribution pattern based on mtDNA sequence data [34] that there was either one large population/refugium with relative constant population size or alternatively that this refugial area included several smaller refugia [34], [35]. However, this issue could not be resolved at the time due to small sample sizes. Thus, several layers of genetic diversity may exist in woodland caribou because of current anthropogenic disturbances and ancient evolutionary processes like the independent evolution of lineages with different adaptive potential. Therefore, comprehensive genetic surveys are warranted to understand the different partitioning of genetic diversity in woodland caribou.

Here, we study the phylogeographical structure of woodland caribou in Canada. More specifically, we focused on woodland caribou populations in Ontario, Manitoba, Saskatchewan, Newfoundland/Labrador, Quebec, and Alberta of which the first three provinces have been notoriously underrepresented in previous studies, but represent major distribution areas for woodland caribou. We surveyed ∼1600 individuals at the mtDNA control region and further supplemented the analyses with published sequences from GenBank [19], [32], [34], [36], [37], [38]. With this approach, we investigated if it is possible to link the phylogeographical structure found in woodland caribou to the last glacial series and date the different lineages. Finally, we discuss the findings of the current study in light of other lines of evidence like the fossil record, physiogeographic, and ecological features in order to describe the most likely scenario for refugial source populations and postglacial migration routes in this subspecies.

## Materials and Methods

### Sampling

Faecal pellets were collected across the range in the winter seasons of 2004–2010. Briefly, aerial transects are flown systematically over caribou ranges within a few days of a snowfall to identify caribou tracks and cratering sites. Following this, sites are accessed by helicopter, faecal samples collected, bagged, kept and shipped frozen to the lab for analysis. In the lab, DNA is extracted from the tissue present in the outer mucosal layer of each sample. Further details for sample collection and lab procedures can be found elsewhere [39]. Samples for mtDNA control region sequencing were selected based on unique microsatellites genotypes generated for other research questions [39], [40], [41]. . Thus, only individuals with different microsatellites genotypes were chosen for sequencing avoiding biasing the analysis by including identical individuals and maximizing haplotype representation. Geographical distribution of samples is shown in Figure S1.

### DNA Extraction

DNA was extracted from the mucosal coat on faecal pellets using a Qiagen DNAeasy tissue extraction kit following manufacturer’s instructions (Qiagen). Briefly, approximately 15 g frozen faecal pellets were thawed and rehydrated in distilled water and subsequently, the mucosal coat was removed using a sterile swab and placed in 500 µl of 1× lysis buffer. Each sample was spiked with 25 µl of proteinase K (Qiagen) and incubated at 65°C for 2 hours followed by a second spike with 25 µl of proteinase K and incubation at 35°C for 12 hours. Each sample was then mixed with an equal volume of AL buffer and incubated at 65°C for 10 minutes. Afterwards, 500 µl of 95% ethanol was added. Each sample was then loaded into a silica column and drawn through using a vacuum pump. Each column was then washed first with 500 µl of AW1 buffer then 500 µl of AW2 buffer. Finally, DNA was eluted using 65 µl of TE buffer heated to 65°C and spinning for 1 minute at 3500*×*g in a microcentrifuge.

### PCR and Sequencing

A 429 bp mtDNA control region fragment was amplified and sequenced using the L15394 and H15947 primers provided by [34] (L15394∶5′ - AAT AGC CCC ACT ATC AGC ACC C- 3′ and H15947∶5′ - TAT GGC CCT GAA GTA AGA ACC AG – 3′). Polymerase chain reactions (20 µl/sample) contained 10 ng of DNA, 1× PCR buffer, 1.5 mM MgCl_2_, 0.2 mM of each dNTP, 2 µM of each primer, 0.4 units of *Taq* polymerase (Invitrogen), and 0.1 mM BSA. PCR conditions were as follows: initial denaturation step of 5 min at 94°C followed by 30 amplification cycles. Each amplification cycle consisted of denaturation for 30 s at 94°C, annealing for 30 s at 56°C, and extension for 30 s at 72°C. A final extension time of 2 min at 72°C was added after completion of amplification cycles. All samples were visualized with ethidium bromide in a 1.5% agarose gel run for 60 minutes at 120 volts. For successfully amplified samples, PCR products were cleaned up using a master mix of 0.90 µl of Antarctic phosphatase buffer, 0.10 µl Antarctic phosphatase, and 0.03 µl exonuclease I (New England BioLabs) per 7 µl of each PCR product and incubated at 37°C for 15 minutes following a second incubation step at 80°C for 15 minutes. For cycle sequencing reactions (12 µl/sample), 1.0 µl of 5× sequencing buffer, 0.5 µl of BigDye Terminator mix v3.1 (Applied Biosystems), 0.8 µM of primer, and 0.5 µl of cleaned PCR product was used. Cycle sequencing conditions were as follows: an initial denaturation step at 96°C for 2 min was followed by 30 amplification cycles consisting of a denaturation step at 96°C for 20 s, annealing at 50°C for 20 s, and an extension at 60°C for 4 min. All sequencing products were run on an ABI 3730 sequencer.

### Quality Checks and Alignment

All sequence data was thoroughly checked by eye and edited manually where necessary using BioEdit [42]. In addition to the generated sequence data for this study, control region sequences from several previous studies [19], [32], [34], [36], [37], [38] were downloaded from GenBank and compiled in a single fasta file. To ensure a high quality alignment and to confidently identify haplotypes, a few sequences including ambiguous sites were discarded. Sequences were trimmed and aligned (using ClustalW implemented in BioEdit) [43] using the program BioEdit [42] and checked by eye afterwards. The program DNaSP v5 [44] was used to identify control region haplotypes and to calculate number of polymorphic sites and nucleotide diversity (Table 1). Subsequently, all unique haplotypes generated in our laboratory were checked another time by eye to confirm sequence quality and haplotype scores.

**Table 1:** Results of demographic analyses for each lineage.

HG	N (H)	N (PS)	π	r	P	R2	P	Fu’s Fs	P
A1	35	27	0.00969	0.043	0.08	0.11575	0.03	−53.395	0.00000
A2	25	17	0.00761	0.090	0.03	0.12779	0.00	−36.086	0.00000
A3	22	21	0.01082	0.026	0.46	0.13073	0.09	−24.586	0.00000

Number of haplotypes N(H) in each haplogroup (HG), number of polymorphic sites N(PS), and nucleotide diversity (π) are given. Harpending’s raggedness (r), Ramos-Onsins & Rozas’ (R2), and Fu’s Fs estimates for the mismatch distributions are shown.

### Phylogenetic Analysis

For maximum-likelihood trees, moose (*Alces alces*, GenBank accession number U12866) was chosen as an outgroup to ensure consistency with the study by [32] and because additional outgroup species (e.g., white-tailed deer and elk) led to the introduction of a high number of gaps, making the alignment unreliable. For maximum likelihood (ML) phylogenetic trees, the program PhyML (version 3.0) [45], [46] was used. The analysis was performed at a high-performance facility (www.bioportal.uio.no). The program jMODELTEST 0.1.1 [47] was used for the statistical selection of the best-fit model of nucleotide substitution. It has been pointed out that hierarchical likelihood ratio tests are not well suited for model selection in phylogenetics, because they are not intended to select from a series of models [48], [49] and favour the more complex model and may thereby lead to overparameterization of the substitution model [50]. Therefore, we used the Akaike information criterion (AIC) [51] to test for the best-fit model of nucleotide substitution. As a result, the GTR+I +G model (gamma shape parameter = 0.32; proportion of invariable sites = 0.48) was identified as the best-fit substitution model. Robustness of ML trees was tested with 2000 bootstrap replicates.

The program BEAST v1.7.2 [52] was used for the Bayesian tree reconstruction using the GTR+I+G substitution model without an outgroup as the inclusion of an outgroup may affect estimation of mutation rates, adherence to a strict molecular clock, and posterior probabilities of an intraspecific phylogenetic reconstruction. The analysis was run twice for 140 million generations and the output was checked in Tracer v1.5 [52] to verify that mixing and convergence of MCMC chains was sufficient. 10% (14 million generations) of the initial samples were removed as burn-in. Effective sample size for all parameters were well above the recommended 200. The tree files for both runs were combined using LogCombiner v1.6.2 [52].

### Network Analysis

However, phylogenetic methods may not lead to the desired resolution at the intraspecific level due to lower genetic diversity and non-hierarchical nature of intraspecific data sets [53], [54] and complementary network approaches might be valuable alternatives to study phylogenetic structure and haplogroups at the population level. Therefore, a network approach might be more appropriate in cases like the caribou, because some of the subspecies/ecotypes are known to be migratory [18] and relationships may be better captured by a network than by a bifurcating tree in this case. Simulations [54] have shown that the median-joining network approach (MJN) [55] outperforms minimum-spanning networks (MSN) because the former is able to infer ancestral haplotypes [54]. Therefore, the program NETWORK 4.6.1.0 [55] was used to reconstruct median-joining trees and networks. Briefly, median-joining trees depict the shortest tree possible (i.e. the tree that connects all haplotypes by the minimum number of steps/mutations) whereas median-joining networks additionally show alternative connections between haplotypes and can be therefore seen as summaries of all shortest trees possible. Importantly, no pruning algorithm was applied to simplify the median-joining network.

To facilitate visualization of spatial distribution of haplotype frequencies and haplogroup proportions, we spatially clustered observations (i.e. geographical locations of samples) using a network-based approach. A graph of sampling locations was constructed using the igraph package for R [56] where graph edges were geographic distances between all pairs of sampling locations. As a threshold, edges greater than 150 km in length were removed to enable clear visualization. This resulted in geographically distinct subgraphs of local sample locations. For these local subgraphs, pie charts of haplotype frequencies and haplogroup proportions were calculated to depict spatial variation in haplogroups and haplotype frequencies in an objective way.

### Calculation of Time to Most Recent Common Ancestors (tMRCA)

Two different methods were applied to date the time to the most recent common ancestor of highly supported lineages identified by phylogenetic analysis. First, we used BEAST v1.7.2 [52] to estimate tMRCAs of highly supported lineages with help of the radiocarbon-dated North American caribou samples by [37], [38] as calibration points. With this approach, it was possible to base the tMRCA calculations on a time calibration window of 0–32600 YBP which encompasses the last glacial maximum (26,500–19,000 YBP).

To check if significant rate heterogeneity existed in the dataset, a first run using an uncorrelated relaxed lognormal molecular clock model was performed. As a result, the UCLD.STDEV parameter values (median = 0.18; lower 95% HPD = 0.0; upper 95% HPD = 0.5) indicated that the strict molecular clock model could not be rejected for this dataset. Consequently, a strict molecular clock model was used to estimate tMRCAs. In order to test if lineages are older than the last glacial maximum and therefore separated and diversified in different glacial refugia, the ‘include stem’ option in BEAST v1.7.2 was used. This option calculates the age of the parent node of the MRCA (i.e., the bottom of the stem leading to that haplogroup). As a second, fundamentally different method, mismatch distributions of pairwise nucleotide differences of each highly supported lineage identified by phylogenetic analysis were calculated. The program Arlequin v3.5 [57] was used to estimate the distribution of the observed pairwise nucleotide site differences (i.e. mismatch distribution, τ) and goodness-of-fit was tested with 10000 bootstrap replicates. The raggedness statistic, r [58] which quantifies the smoothness of the observed pairwise difference distribution (population growth = lower *r* values) was also calculated in Arlequin v3.5 [57]. A smooth morphology indicates that the population in question has undergone an expansion [58] whereas a ‘ragged’ morphology indicates a relative constant population size. Since the raggedness statistic is considered to have low power to detect population expansions [59] two additional statistics, Fu’s Fs [60] and Ramos-Onsins and Rozas’s R2 [59] were calculated in DNaSP v5 [44]. Mismatch distribution analysis are ideally suited for long-ranging species [61] and therefore are likely to be appropriate to use in large mammals like caribou. Previous studies [19], [62], [63] have pointed out that evolutionary rates are greatly underestimated at the intraspecific level leading potentially to an overestimation of expansion and divergence times. Therefore, an evolutionary rate of 58.9%/million years based on the bovine control region [19], [64] was chosen for the calculation of expansion times in caribou.

## Results

### Summary Statistics

In total, 252 mtDNA control region haplotypes among 1917 caribou samples could be identified. For woodland caribou, 135 haplotypes could be identified among 1655 samples of which 51 haplotypes are reported for the first time (GenBank accession numbers XXX-XXX). Notably, these numbers include some Yukon locations (e.g. Watson Lake [32]) that may need taxonomic revision and therefore, these numbers are preliminary. Excluding gaps resulted in only one change: H156 was identical to 119. Further information regarding spatial distribution of haplotypes including frequencies can be found in Table S1.

### Phylogenetic and Network Analysis

**Figure 1:**
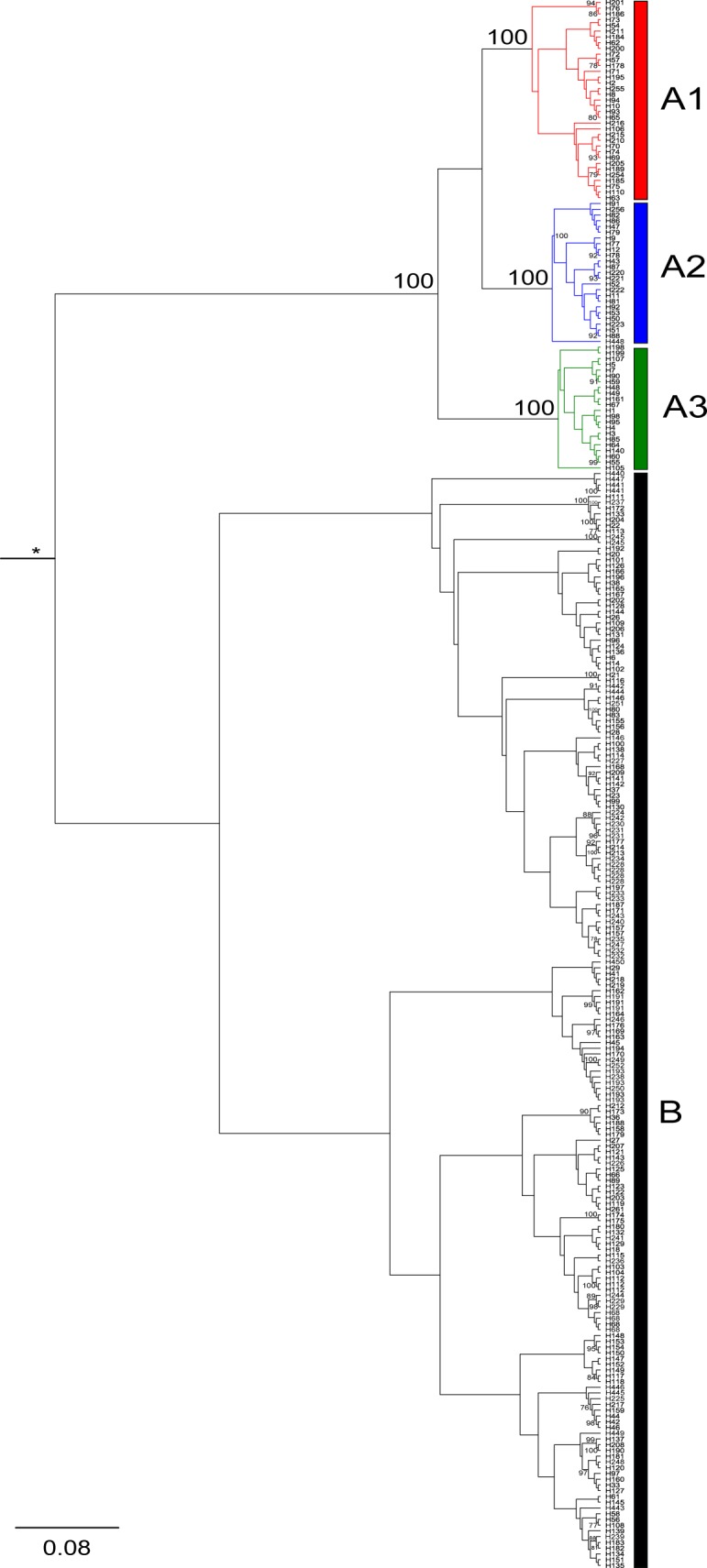
Bayesian phylogenetic tree reconstruction. Bayesian phylogenetic tree reconstruction using mitochondrial control region haplotypes. Bayesian posterior probability (≥ 75%) are shown. Coloured branches represent haplogroups (red = A1, blue = A2, green = A3). The branch labelled with an * is shortened by 90%.

**Figure 2:**
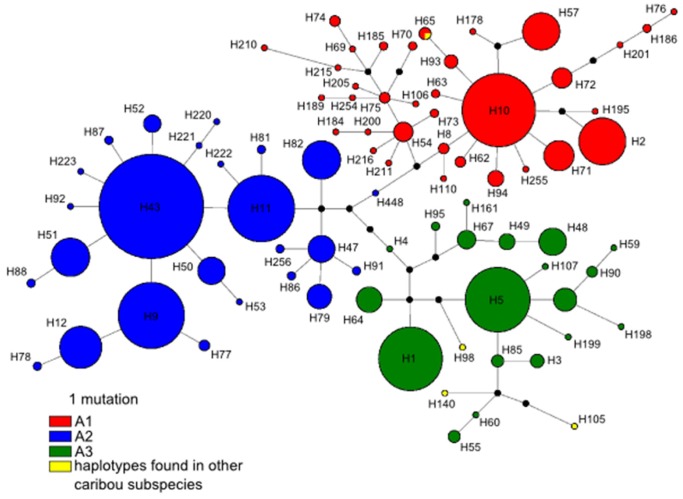
Median-joining tree. Median-joining tree of the three mtDNA control region haplogroups A1–A3 identified in this study. Circles represent haplotypes and circle size is proportional to haplotype frequencies. Circles are coloured according to haplogroup membership: A1 = red; A2 = blue, A3 = green, and yellow = haplotypes that are found in other caribou subspecies.

The Bayesian phylogenetic tree reconstruction clearly identified two major haplogroups in caribou across Canada separating woodland caribou (haplogroup A) from other caribou subspecies (haplogroup B) indicating that the woodland caribou most likely originated from a distinct origin south of the Laurentide ice sheet [14], [32]–[34] whereas the other four (arctic/tundra) subspecies originated in northern refugia. Importantly, the Northern Mountain caribou ecotype which is currently included in the woodland caribou subspecies was found to be more closely related to B haplotypes. Consequently, the Northern Mountain caribou ecotype may have originated from one of the northern refugia and a taxonomic revision of this specific ecotype may be required [65]. Interestingly, haplogroup B consisted of 170 haplotypes (474 individuals) whereas the distinct haplogroup A consisted of 82 haplotypes although far more A haplogroup caribou samples (1443 individuals) were analysed. Out of these 82 haplotypes found in haplogroup A, 79 (96%) were found only or in a very high proportion in woodland caribou; thereby clearly separating woodland caribou from all other subspecies (Fig. 1 & Figure S2) whereas three haplotypes (H98, H105, and H140) were only found in Peary and barren-ground caribou in single individuals, respectively (Fig. 2). Given the otherwise clear separation of woodland caribou from other caribou subspecies, the most likely explanation is that these three haplotypes were introduced to the other subspecies by introgression. Cluster A consisted of three well-supported subhaplogrops (A1, A2, and A3) based on the Bayesian tree reconstruction. The ML tree reconstruction supported the distinction of the two major haplogroups (A and B, Figure S3) and recovered also the three haplogroups A1–A3. However, bootstrap values were generally low, which can be probably best explained by the fact that these lineages were separated by only a few point mutations as revealed by the median-joining tree and network (Fig. 2 & Figure S4) and that the bootstrap analysis is considered to be conservative [66].

The median-joining tree (Fig. 2) further revealed that the lineages A1–A3 have specific central haplotypes (A1: H10, H54, and H75; A2: H43 and H47; A3: H1 and H5) from which all of the other haplotypes found in the respective lineage are derived by mutations. Notably, these central haplotypes were also found in high frequencies. These patterns are expected by coalescent theory predictions [53] and indicate that these haplotypes are ancestral. Importantly, no connections between haplotypes from different haplogroups were found (Figure S4), thereby further supporting the distinctiveness of lineages.

**Figure 3:**
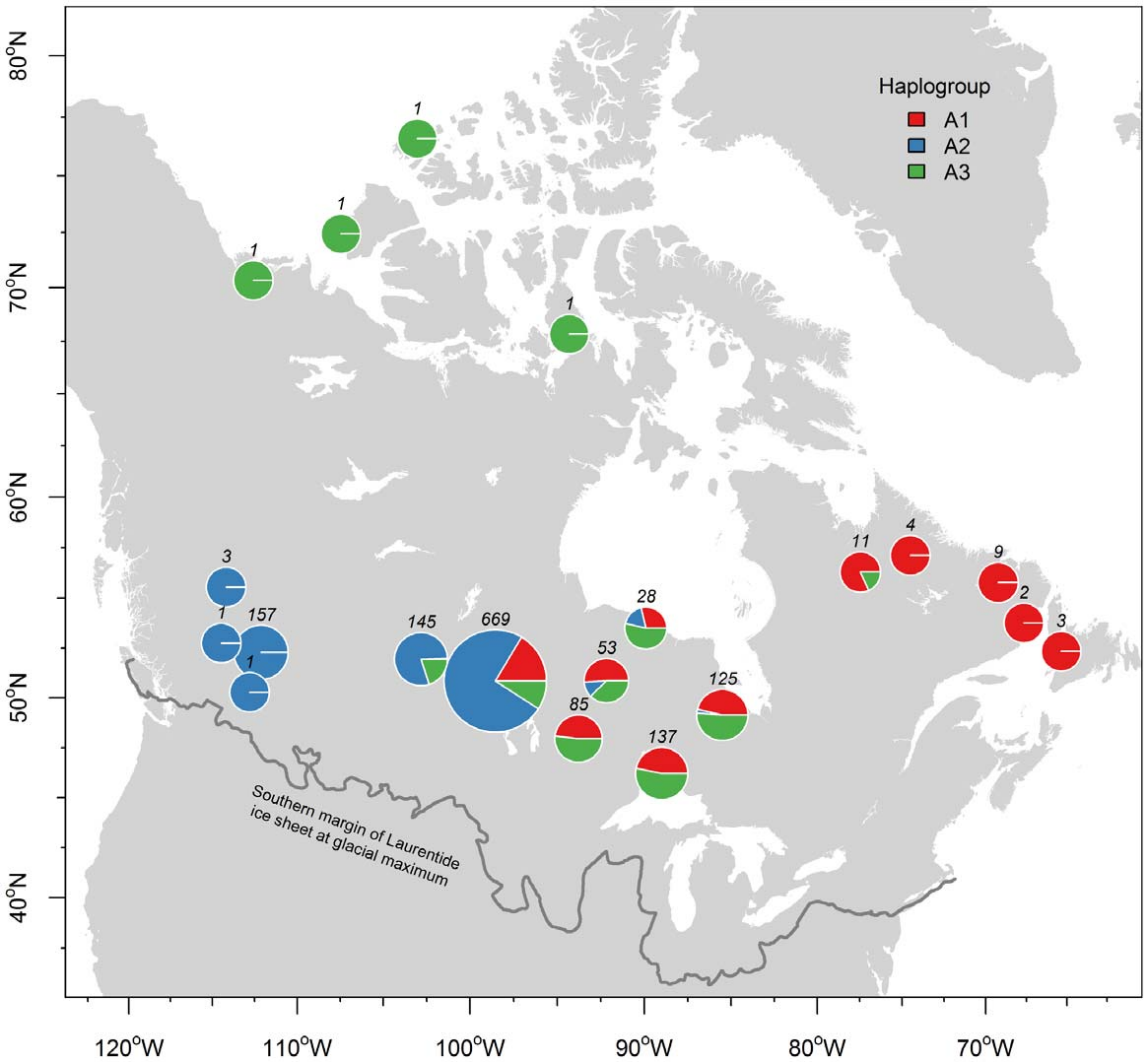
Geographical distribution of lineages A1–A3. Current spatial distribution of the three identified lineages A1 (red), A2 (blue), and A3 (green). The maximum extension of the Laurentide ice sheet is given as a solid grey line.

**Figure 4:**
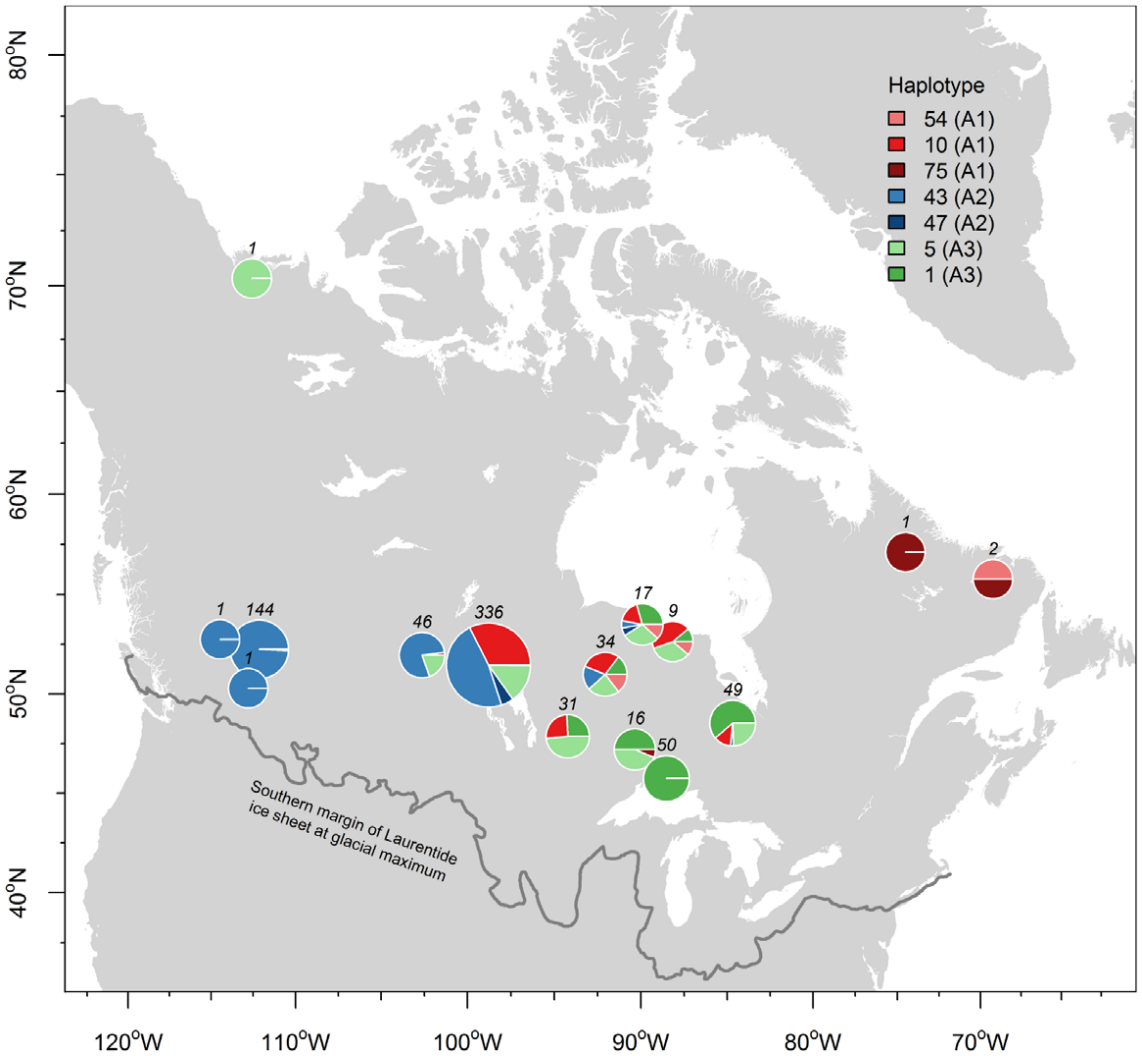
Geographical distribution of central haplotypes of lineages A1–A3. Current spatial distribution of central haplotypes in lineages A1 (red shading: H10, H54, and H75), A2 (blue shading: H43 and H47), and A3 (green shading: H1 and H5). The maximum extension of the Laurentide ice sheet is given as a solid grey line.

When plotting individuals according to their haplogroup membership on a map (Fig. 3), a gradual separation of lineages became apparent with A2 haplotypes mainly distributed in the Western part of Canada whereas haplogroups A1 and A3 had overlapping distribution ranges in Ontario. Notably, A3 haplotypes were found only in a few individuals in the Eastern part of Canada. Additionally, we plotted the geographical distribution of ancestral haplotypes for each haplogroup (A1–A3; Fig. 4). This revealed that the majority of individuals carrying A3 ancestral haplotypes were found in (Southern) Ontario whereas the majority of individuals carrying A1 ancestral haplotypes were found in Manitoba. However, it should be noted that our sampling was much lower in the Eastern part of Canada (i.e. Quebec, Newfoundland, and Labrador) and therefore, additional sampling in these areas is required to clarify if the low representation of haplogroup A3 in this region is a real pattern or a sampling artefact. Likewise, increased sampling might lead to altered frequencies of A1 (ancestral) haplotypes in this region. It is, however, unlikely that a lot more new haplotypes will be found because the median-joining tree (Fig. 2) appears to be almost complete. Therefore, it is also highly improbable that additional major glacial refugia were missed.

### tMRCA and Dating of Sudden Expansion

**Figure 5:**
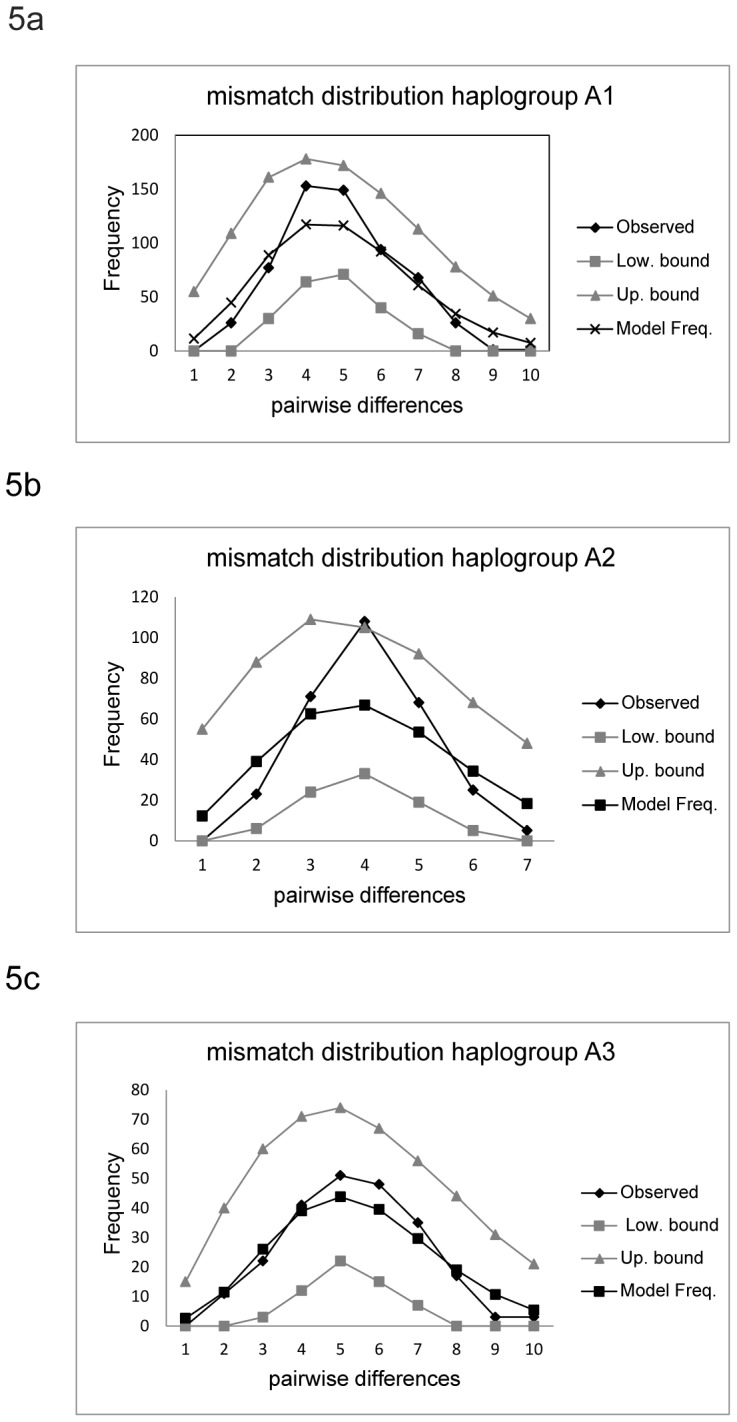
a–c. Mismatch distributions of lineages A1–A3. Mismatch distribution of pairwise nucleotide differences of mtDNA control region sequences among individuals within haplogroups A1 (Fig. 5a), A2 (Fig. 5b), and A3 (Fig. 5c). Figures show observed values of number of pairwise differences, model frequencies under a sudden expansion model, and lower and upper bounds of model frequencies.

The mismatch distribution analysis strongly supported a sudden expansion model in the three woodland caribou subhaplogroups A1–A3 (Table 1, Fig. 5a–c). Fu’s Fs estimates were highly significant in all three cases while R2 values [59] were significant in two cases (A1 & A2) and were close to significance in the third case (A3). The less powerful Harpending’s raggedness index (r) was significant in one case (A2). All three subhaplogroups showed typical unimodal distributions (Fig. 5a–c) expected under a sudden expansion model. The time of these expansions was dated to 16660 (A1; 95% CI: 9285–22897), 13544 (A2; 95% CI: 5491–20369), and 18921 (A3; 95% CI: 8849–27114) years before present (YBP, Table 2) using the mean number of pairwise differences (τ). In contrast, applying the Bayesian method to date the tMRCA of the three highly supported lineages resulted in consistently older estimates (A1∶20072 YBP [95% CI: 10009–32281]; A2∶15627 YBP [95% CI: 6752–25713]; and A3∶22005 YBP [95% CI: 9789–38854]). The Bayesian method might be a bit more accurate because of the radiocarbon-dated sample calibration. Nevertheless, both methods resulted in comparable estimates for the MRCA that indicated postglacial expansions immediately after the retreat of the Laurentide ice sheet for lineages A1 and A3. Lineage A2 appears to have undergone a postglacial expansion somewhat later. However, the tMRCA estimates including the stem (i.e. calculation of the age of the parent node of the MRCA or the bottom of the stem leading to that clade) for the three lineages (Table 2, A1∶40030, 95% CI: 25302 – 62211; A2∶38130, 95% CI: 25424–56034; A3∶47950, 95% CI: 25708–77391) indicated that all three lineages diverged before the last glacial maximum. Therefore, it seems to be highly likely that all lineages came from separate glacial refugia.

**Table 2:** Molecular dating of expansions and tMRCA.

HG	τ- mean	CI (95%)	demographic expansion date (YBP)	CI (95%)	tMRCA (YBP)	CI (95%)	tMRCA (YBP) including stem	CI (95%)
A1	3.935	2.193–5.408	16660	9285–22897	20072	10009–32281	40030	25302–62211
A2	3.082	1.137–4.701	13544	5491–20369	15627	6752–25713	38130	25424–56034
A3	4.469	2.090–6.404	18921	8849–27114	22005	9789–38854	47950	25708–77391

The average number of pairwise differences (Tau, τ) and confidence intervals (95% CI) were calculated with DNaSP v5 (Librado and Rozas 2009) for each haplogroup (HG). Expansion dates are given for the mean and the 95% CI. The tMRCA and 95% CI were calculated with BEAST v1.7.2 (Drummond and Rambaut 2007). YBP = years before present.

## Discussion

Our analysis clearly identified two major haplogroups in caribou across Canada separating woodland caribou from other caribou subspecies. This is in congruence with other studies [19], [34], [65]. In the first lineage (A), three highly supported lineages (A1–A3) could be identified. All of these three lineages likely underwent sudden expansions ∼15600–22000 YBP as indicated by the Bayesian molecular clock analysis. Importantly, the three lineages likely diverged before the last glacial maximum (38130–47950 YBP) suggesting that these lineages are representatives of three geographically separated full glacial refugia.

Given that the majority of woodland caribou haplotypes were assembled into lineages A1–A3, the phylogeographical structure within woodland caribou in that geographical region appears to be mainly caused by postglacial expansions after the last glacial maximum. The spatial distribution of lineages A1–A3 haplotypes (Fig. 3) supports a scenario of at least two, most likely three, *geographically* well-separated refugial origins. For example, if there would have been only one glacial refugium or three refugia in one geographically restricted area, one would expect that lineages would have mixed early on in the expansion and subsequently would have spread throughout Canada with relatively similar haplotype proportions. This is clearly not the case (Fig. 3 & 4) since there is a gradual separation from West to East, although there are overlapping zones in Manitoba and Ontario. These overlapping zones are expected, given that woodland caribou is highly mobile [18].

The fossil record shows that caribou was found in West Virginia (New Trout Cave 17060–28250 YBP) [33], Tennessee (three caves in Sullivan county at least 20000 years old) [33], and in the Appalachian Mountains (16500–20500 YBP) several hundred kilometres south of the Laurentide ice sheet [33]. It was also pointed out by [33] that of 42 dated caribou fossils, 41 were found 300–800 km south of the tundra zone of that period, indicating that southern glacial refugia likely existed for caribou. Thus, one major concentration of caribou before 13000 YBP was found in the Appalachians [33] in congruence with findings by [10] that this region served as a glacial refugium for plant and animal species. However, the rather clear geographical separation of haplogroup A2 (Fig. 3, in blue) with a distribution centre in Western Canada, point to another geographically well-separated glacial refugium. Caribou remains have been found also in NW Alabama (11820 YBP) [67] and on the border Illinois/Missouri [14], [30] west of the Apalachicola River system /Appalachian Mountains and in Southern Idaho [68] west of the Mississippi River linked to the Rocky Mountains. Hence, the fossil record indicates that multiple glacial refugia are probable; given the occurrence of caribou remains throughout the US [14].

From an ecological and physiogeographic perspective, the Rocky Mountains in the western USA and the Appalachian Mountains in the eastern part of the US have been suggested as potential southern glacial refugia for the woodland caribou [14], [33]. It has been hypothesized [33] that Pleistocene caribou adapted to the climate in the Appalachian Mountains, probably to avoid predator pressure in the lowlands. Similarly, [14] suggested – based on the fossil record - that woodland caribou might have been distributed ‘*in a tundra belt across the southern edge of the ice-sheet from New Jersey, Kentucky, Missouri, Illinois, Iowa to the mountainous region of the southwest - New Mexico and Nevada’* and may have survived the last glacial maximum in the mountainous regions of New Mexico and Nevada because of the tundra-like habitat. Thus, from a physiogeographic/ecological perspective, the Rocky Mountains in the West and the Appalachian Mountains in the East are the most likely glacial refugia for woodland caribou. However, a third lineage of which the most ancestral haplotypes were found mainly in Manitoba, most likely came from east of the Mississippi, but west of the Appalachians. This would explain the gradual separation of these three haplogroups from west to east (Fig. 3 & Fig. 4). Also, the high proportion of the central haplotypes H10 found in Manitoba suggests that the centre of lineage A1 was somewhere south of Manitoba. In North America, multiple glacial refugia have been identified in the Gulf of Mexico coastal region separated by the Mississippi River and the Apalachicola River /Appalachian mountain system for different animal and plant species [10], [13]. For example, mammal species like the short-tailed shrew (*Blarina brevicauda*) [69] exhibit most likely three glacial refugial origins: one west of the Mississippi and two refugia in the southern Appalachians. Also, it could be shown that highly mobile species like the white-tailed deer (*Odocoileus virginianus*) [70] show similar phylogeographical differentiation east and west of the Apalachicola River, thus probably originating from two glacial refugia. Multiple refugia could be also found in the North American mule deer (*Odocoileus hemionus*) [71], another large, mobile ungulate. Therefore, we propose that a third glacial refugium for woodland caribou was likely situated west of the Appalachian Mountains and east of the Mississippi River. The latter may have acted as a guiding corridor for a northwards migration and prevented mixing with the western lineage (A2) to a certain extent. Some authors [72] postulated that there have been two eastern glacial refugia including one in Wisconsin south of the ice sheet. This seems probable if caribou migrated eastwards into Manitoba. In this case, the Great Lakes may have acted as a physiogeographic barrier for a westward movement into Ontario and today’s mixing of haplogroups A1 and A3 in Ontario is due to bidirectional movement at a later point of time. Thus, there is a possibility that lineages A1 and A3 came from two geographically separated eastern refugia in Wisconsin and in the Appalachians and A2 originated in the Rocky Mountains. Importantly, a third refugium south of Manitoba would not be associated with a mountain chain, suggesting possibly different ecological adaptations of this lineage. Finally, [5] showed that highly mobile species and/or species with large contemporary distribution ranges are significantly more likely to have been located in multiple glacial refugia during the glacial series in North America. These findings support the existence of multiple glacial refugia in the woodland caribou, given that this subspecies is highly mobile and has extensive contemporary ranges.

To conclude, different lines of evidence (fossil record, ecological considerations, physiogeography) point to glacial refugia in the Rocky Mountains and the Northern/Central Appalachian Mountains for woodland caribou. This is in congruence with our genetic data set that showed a clear ‘western’ clade (A2, Fig. 3 & Fig. 4) that most likely originated from the Rocky Mountains. Similarly, another glacial refugium presumably originated in the Appalachian Mountains in the east and then migrated north into Ontario (A3, Fig. 3 & Fig. 4) and possibly Quebec. We predict that with better sampling from (Southern) Quebec, more lineage A3 haplotypes will be found there. This would correspond well with previously suggested migration routes [32] that there was a glacial refugium south of the Laurentide ice sheet and that northwards migrating caribou split routes somewhere north of the Great Lakes area and migrated into Quebec on one hand and Ontario on the other. The Northern Great Lakes area was covered by the Laurentide ice sheet until ∼10000 YBP [73] and therefore, the split of this refugial population must have occurred less than 10000 years ago. A likely third glacial refugium west of Appalachian Mountains and east of the Mississippi River was suggested by the phylogenetic analysis (Fig. 1) and the gradual geographical distribution of the central ancestral haplotypes (Fig. 4).

The identification of expansions from glacial refugia for caribou presented here should not be seen in isolation but hopefully nurture further scientific investigations to characterize ecological and microclimatic parameters of these refugial lineages and facilitate eventually the designation of meaningful conservation units for this threatened caribou subspecies. The importance of an integrative approach for the characterization of refugia has been pointed out by others [5], [7] and will increase our understanding of evolutionary processes and individual species responses to climate changes.

## Supporting Information

Figure S1 **Distribution map of different caribou subspecies and samples included in this study.** GenBank samples (white circles) and samples sequenced for this study (black circles) are shown.
(PNG)

Figure S2 **Bayesian phylogenetic tree reconstruction based on mitochondrial control region haplotypes.** Bayesian posterior probabilities (>75%) are shown. Coloured branches represent haplogroups (red = A1, blue = A2, green = A3). The branch labelled with an * is shortened by 90% and haplotype names are given.
(PDF)

Figure S3 **Maximum-Likelihood tree of mtDNA control region haplotypes showing the two major haplogroups (A and B) including the three ancient lineages in haplogroup A (A1–A3).** Bootstrap values > 40% are shown.
(PDF)

Figure S4 **Median-joining network of the three identified lineages (A1–A3) in woodland caribou.** Circles represent haplotypes and circle size is proportional to haplotype frequencies. Circles are coloured according to haplogroup membership: A1 = red, A2 = blue, A3 = green, and yellow = haplotypes that are found in other caribou subspecies.
(PDF)

Table S1 **Haplotype list including the longitudes and latitudes of the pie charts in** **figure 3** **.**
(XLS)
