# Supplementary figures and images for: Phylogeographical Analysis of mtDNA Data Indicates Postglacial Expansion from Multiple Glacial Refugia in Woodland Caribou (Rangifer tarandus caribou)
Source: PLoS One. 2012 Dec 21;7(12):e52661. doi: 10.1371/journal.pone.0052661 (PMC3528724; doi:10.1371/journal.pone.0052661)

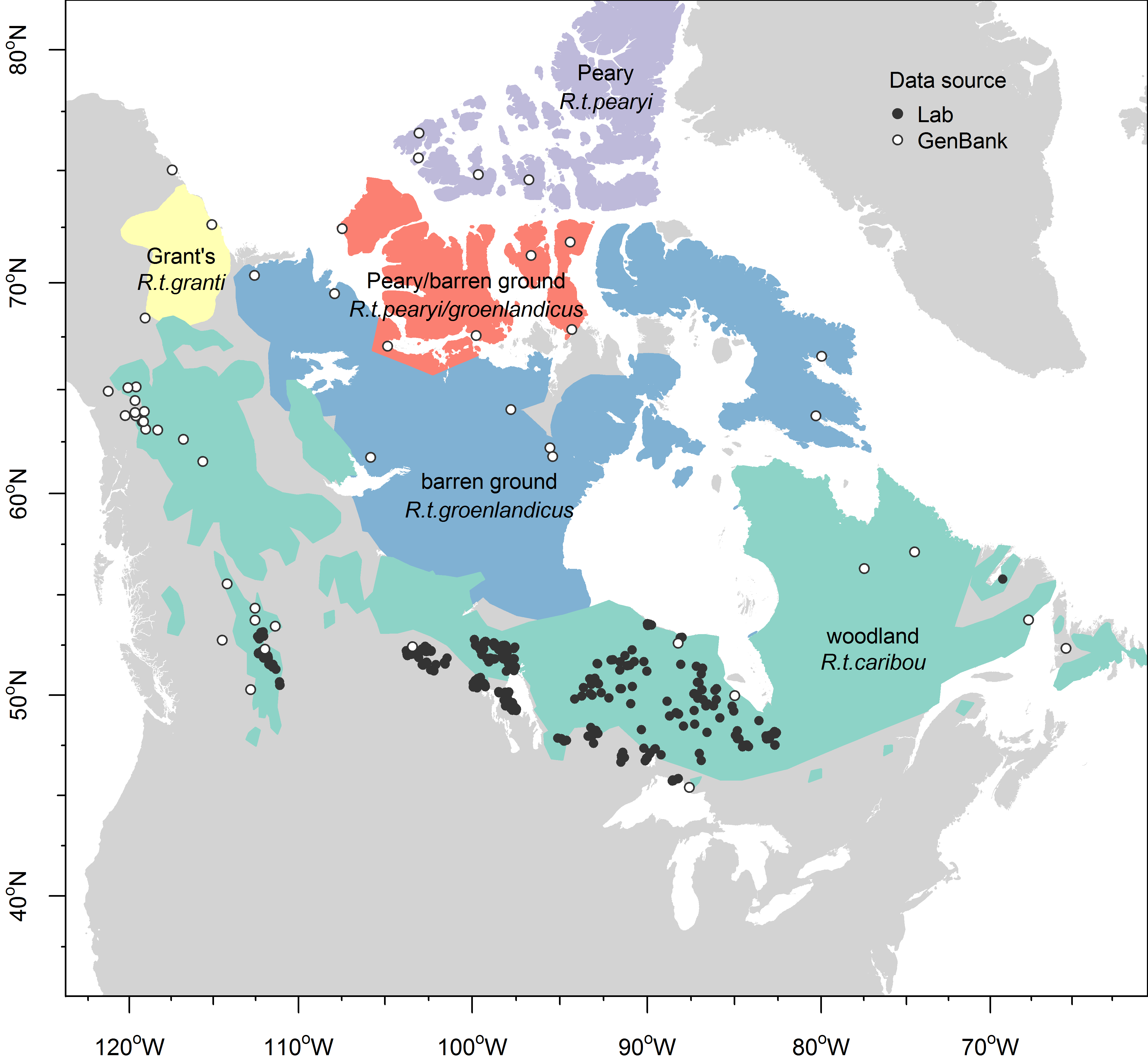

Supplement: Figure S1 — Distribution map of different caribou subspecies and samples included in this study. GenBank samples (white circles) and samples sequenced for this study (black circles) are shown. (PNG) [file pone.0052661.s001.png]
