# Supplementary material for: Phylogeographical Analysis of mtDNA Data Indicates Postglacial Expansion from Multiple Glacial Refugia in Woodland Caribou (Rangifer tarandus caribou)
Source: PLoS One. 2012 Dec 21;7(12):e52661. doi: 10.1371/journal.pone.0052661 (PMC3528724; doi:10.1371/journal.pone.0052661)
Supplement: Figure S2 — Bayesian phylogenetic tree reconstruction based on mitochondrial control region haplotypes. Bayesian posterior probabilities (>75%) are shown. Coloured branches represent haplogroups (red = A1, blue = A2, green = A3). The branch labelled with an * is shortened by 90% and haplotype names are given. (PDF) [file pone.0052661.s002.pdf]

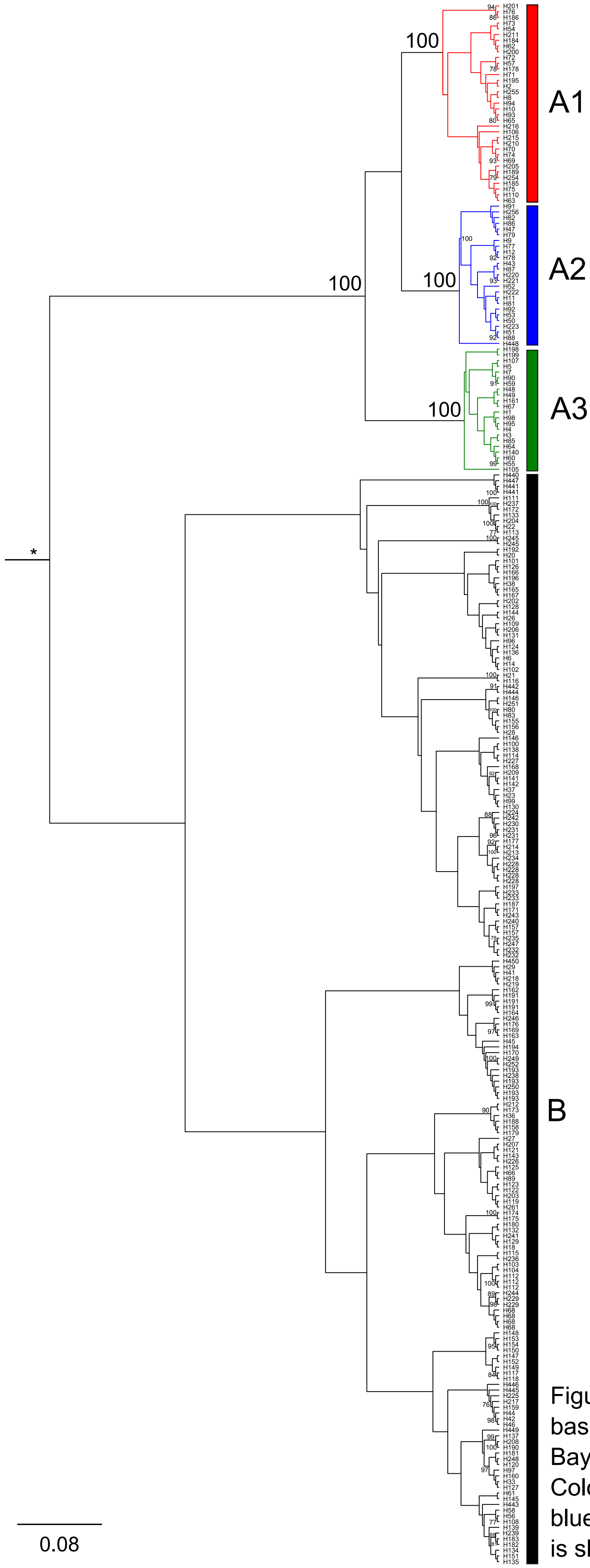

Figure S2: Bayesian phylogenetic tree reconstruction based on mitochondrial control region haplotypes. Bayesian posterior probablities (>75%) are shown. Coloured branches represent haplogroups (red = A1, blue = A2, green = A3). The branch labelled with an * is shortened by 90% and haplotype names are given.
