# Supplementary material for: Phylogeographical Analysis of mtDNA Data Indicates Postglacial Expansion from Multiple Glacial Refugia in Woodland Caribou (Rangifer tarandus caribou)
Source: PLoS One. 2012 Dec 21;7(12):e52661. doi: 10.1371/journal.pone.0052661 (PMC3528724; doi:10.1371/journal.pone.0052661)
Supplement: Figure S3 — Maximum-Likelihood tree of mtDNA control region haplotypes showing the two major haplogroups (A and B) including the three ancient lineages in haplogroup A (A1–A3). Bootstrap values > 40% are shown. (PDF) [file pone.0052661.s003.pdf]

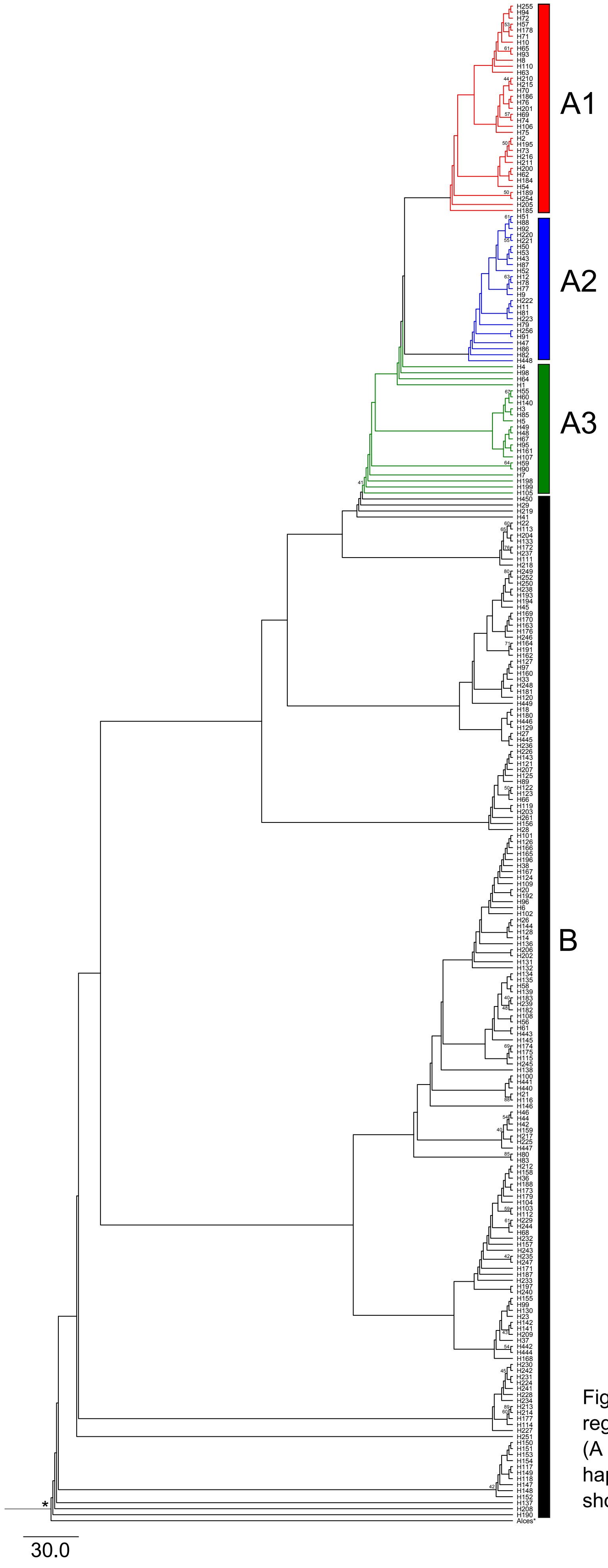

Figure S3. Maximum-Likelihood tree of mtDNA control region haplotypes showing the two major haplogroups (A and B) including the three ancient lineages in haplogroup A (A1-A3). Bootstrap values > 40% are shown. The root (*) is shortened by 90%.
