# Supplementary material for: Phylogeographical Analysis of mtDNA Data Indicates Postglacial Expansion from Multiple Glacial Refugia in Woodland Caribou (Rangifer tarandus caribou)
Source: PLoS One. 2012 Dec 21;7(12):e52661. doi: 10.1371/journal.pone.0052661 (PMC3528724; doi:10.1371/journal.pone.0052661)
Supplement: Figure S4 — Median-joining network of the three identified lineages (A1–A3) in woodland caribou. Circles represent haplotypes and circle size is proportional to haplotype frequencies. Circles are coloured according to haplogroup membership: A1 = red, A2 = blue, A3 = green, and yellow = haplotypes that are found in other caribou subspecies. (PDF) [file pone.0052661.s004.pdf]

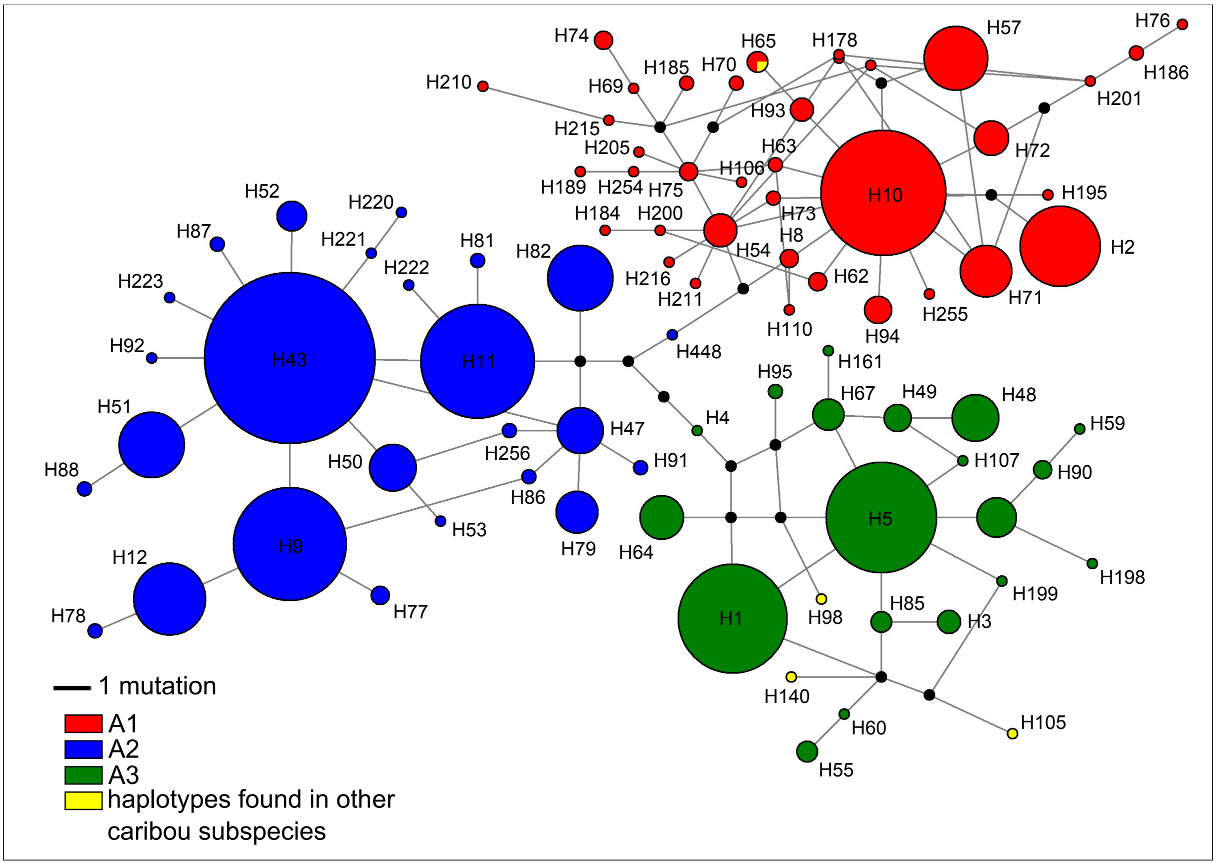

Figure S4. Median-joining network of the three identified lineages (A1-A3) in woodland caribou. Circles represent haplotypes and circle size is proportional to haplotype frequencies. Circles are coloured according to haplogroup membership: A1 = red, A2 = blue, A3 = green, and yellow = haplotypes that are found in other caribou subspecies.
